# Risk of Chronic Obstructive Pulmonary Disease Exacerbation in Patients Who Use Methotrexate—A Nationwide Study of 58,580 Outpatients

**DOI:** 10.3390/biomedicines9060604

**Published:** 2021-05-26

**Authors:** Christina Marisa Bergsøe, Pradeesh Sivapalan, Mohamad Isam Saeed, Josefin Eklöf, Zaigham Saghir, Rikke Sørensen, Tor Biering-Sørensen, Jens-Ulrik Stæhr Jensen

**Affiliations:** 1 Department of Medicine, Section of Respiratory Medicine, Herlev and Gentofte Hospital, University of Copenhagen, 2900 Hellerup, Denmark; christina.marisa.bergsoee@regionh.dk (C.M.B.); mohamad.isam.saeed.02@regionh.dk (M.I.S.); josefin.viktoria.ekloef@regionh.dk (J.E.); zaigham.saghir@regionh.dk (Z.S.); jens.ulrik.jensen@regionh.dk (J.-U.S.J.); 2 Department of Internal Medicine, Zealand University Hospital, 4000 Roskilde, Denmark; 3 Department of Clinical Medicine, Faculty of Health and Medical Sciences, University of Copenhagen, 2200 Copenhagen, Denmark; 4 Department of Cardiology, Copenhagen University Hospital Rigshospitalet, 2100 Copenhagen, Denmark; rikke.soerensen@regionh.dk; 5 Department of Cardiology, Herlev and Gentofte Hospital, University of Copenhagen, 2900 Hellerup, Denmark; tor.biering-soerensen@regionh.dk; 6 Department of Infectious Diseases, Rigshospitalet, University of Copenhagen, 2100 Copenhagen, Denmark

**Keywords:** chronic obstructive pulmonary disease, exacerbation of chronic obstructive pulmonary disease, anti-inflammatory, airway inflammation, immunosuppression

## Abstract

Patients with severe chronic obstructive pulmonary disease (COPD) experience frequent acute exacerbations and require repeated courses of corticosteroid therapy, which may lead to adverse effects. Methotrexate (MTX) has anti-inflammatory properties. The objective of this study was to describe the risk of COPD exacerbation in patients exposed to MTX. In this nationwide cohort study of 58,580 COPD outpatients, we compared the risk of hospitalization-requiring COPD exacerbation or death within 180 days in MTX vs. non-MTX users in a propensity-score matched study population as well as an unmatched cohort, in which we adjusted for confounders. The use of MTX was associated with a reduction in risk of COPD exacerbation in the propensity-score matched population at 180 days follow-up (HR 0.66, CI 0.66–0.66, *p* < 0.001). Similar results were shown in our sensitivity analyses at 180-day follow-up on unmatched population and 365-day follow-up on matched and unmatched population (HR 0.76 CI 0.59–0.99, HR 0.81 CI 0.81–0.82 and HR 0.92 CI 0.76–1.11, respectively). MTX was associated with a lower risk of COPD exacerbation within the first six months after study entry. The finding seems biologically plausible and could potentially be a part of the management of COPD patients with many exacerbations.

## 1. Introduction

Chronic obstructive pulmonary disease (COPD) is a disease characterized by difficulty in breathing, impaired lung function and frequent lung infections. The precise global prevalence of COPD is unknown, but it is estimated to be between 251 and 384 million people worldwide [1,2]. The disease is associated with increased morbidity and mortality with recurrent acute worsening of COPD symptoms (COPD exacerbation) as the leading cause of mortality in COPD patients [3].

Patients with acute exacerbation of COPD are administered systemic corticosteroids to decrease the inflammation that usually occurs during COPD exacerbation [4]. Even though COPD exacerbation can generally be effectively treated with corticosteroids, bronchodilators and antibiotics, not infrequently an acute exacerbation destabilizes the patient which may lead to progressive respiratory failure and eventually, death. Often this happens in COPD patients with long-standing chronic or recurrent inflammation despite guideline-based management and therefore, for this high-risk group in which a large part of the COPD-related mortality happens, other stabilizing therapeutics are needed. Systemic corticosteroids in lower doses as such stabilizing therapy may be a tempting option, but used continuously, corticosteroids are also associated with many side effects including increased risk of pneumonia, osteoporosis [5], cataracts [6], mycobacterial infection [7], diabetes [8,9] and others.

Methotrexate (MTX) is an immunosuppressive agent that inhibits the cell’s DNA synthesis, thus, among other effects, reducing cell replication [10]. This property of MTX inhibits the proliferation of immune cells during inflammation and produces an anti-inflammatory effect. The anti-inflammatory properties of MTX can also be attributed to the release of adenosine and anti-inflammatory cytokines coupled with the inhibition of proinflammatory cytokines [11].

MTX is administered in the treatment of several systemic inflammatory diseases such as rheumatoid arthritis, systemic lupus erythematosus, psoriasis and atopic dermatitis [12,13]. The use of MTX in disease control and as a corticosteroid-sparing agent has also been observed in sarcoidosis [14] and asthma patients [15,16,17]. Unlike sarcoidosis and asthma, MTX has not been studied to treat COPD as an anti-inflammatory agent or corticosteroid-sparing agent. We aim to investigate whether MTX is associated with reduced risk of acute hospitalization-requiring COPD exacerbation (AECOPD) and thereby might have the potential as a possible corticosteroid-sparing drug in COPD patients.

The objective of this study was to determine the effect of MTX on the risk of acute hospitalization-requiring exacerbation of COPD (AECOPD) in patients with moderate to severe COPD with all-cause mortality as competing event. We addressed this question using a nationwide series of patients diagnosed with COPD with and without MTX treatment.

We hypothesized that COPD patients who use MTX for systemic inflammatory diseases are at lower risk of AECOPD compared to COPD patients who do not use MTX.

## 2. Materials and Methods

### 2.1. Study Subjects

This observational cohort study had a population consisting of COPD patients with outpatient clinic visit whose clinical records were available in the Danish Registry of Chronic Obstructive Pulmonary Disease (DrCOPD). Inclusion criterium was COPD diagnosis verified by a respiratory medicine specialist and spirometry. Exclusion criteria were patient age <40 years and sarcoidosis diagnosis. Patients meeting study criteria were classified into two cohorts (MTX user vs. non-MTX user). The exposure group was COPD patients that receive MTX treatment, and the control group was COPD patients who did not receive MTX treatment. We defined MTX users as patients with collection of at least one MTX prescription within two years prior to study entry. Treatment with MTX more than two years prior to study entry or after study entry was not considered as MTX exposure in this study.

### 2.2. Study Design

The period of inclusion was from 1 January 2010 until 31 December 2017 with the study entry being the day the patients enter DrCOPD (i.e., first outpatient clinic visits registered). Patients were followed from date of study entry until the occurrence of COPD exacerbation, death or 31 December 2017.

Data is collected and reviewed with the help of an online database administered by The Danish Health Data Authority. Due to the register-based nature of this study, it was not necessary to obtain informed consent from study participants according to the Danish legislation. Patients or the public were not involved in the design, conduct, reporting or dissemination plans of our research.

The outcome of interest in this study was the presence of AECOPD within 180 days from study entry.

### 2.3. Data Sources

Data obtained from DrCOPD were linked to data from multiple nationwide registries through a unique personal identification number given at birth or immigration. The following registries were used in this study:

- The Danish Registry of Chronic Obstructive Pulmonary Disease (DrCOPD) contains information on treatment and health status of all patients with COPD in Denmark. Data obtained from this registry included age, lung function reported as expected forced expiratory volume in 1 s (FEV1) % predicted, body mass index (BMI), smoking status and vital status.
- The Danish National Health Service Prescription Database contains information on all prescriptions including the date of dispensation, the dose dispensed and the strength of prescriptions. Data was coded according to the Anatomical Therapeutic Chemical (ATC) classification system (Table S1). Medications included were MTX, inhaled corticosteroids (ICS), long-acting muscarinic antagonist (LAMA), long-acting beta-agonist (LABA) and oral corticosteroids (OCS).
- The Danish National Patient Register contains information on hospital admissions and outpatient clinic visits in Denmark. The primary and secondary diagnoses from each hospital visit were coded according to the International Classification of Diseases, 10th revision Clinical Modification Code (ICD-10) (Table S2). The diagnoses included were sarcoidosis, asthma, heart failure, ischemic heart disease, diabetes, peptic ulcer, dementia, solid metastatic tumor, hemiplegia or paraplegia, renal failure, peripheral vascular disease, cerebrovascular disease, rheumatic disease, any malignancy except malignant neoplasm of skin, mild liver disease, moderate to serious liver disease and AECOPD.

### 2.4. Analyses

For the initial summarization of data, categorical variables were expressed as number of observations with percentages. Variables that were continuously distributed were categorized into groups (BMI group, age group, Global Initiative for Chronic Obstructive Lung Disease (GOLD) class) and expressed as medians and interquartile range (IQR). Categorical variables were studied using chi-square test. Cox-proportional hazard model and cumulative incidence function were used for comparison of exacerbation risk in the group receiving MTX treatment and the group not receiving MTX treatment. Log-rank test provided a statistical comparison of cumulative incidence curves of the two study cohorts. We performed the analysis with all-cause mortality as competing risk.

The main analysis was performed on the propensity score matched population. The matching of the population was done using Greedy matching algorithm with a 1:10 matching ratio [18]. Patients were matched using characteristics that may increase their risk of AECOPD namely GOLD stage 1–4 (Lung function (FEV_1_%); Stage 1: >80%, Stage 2: 50–79.99%, Stage 3: 30–49.99%, Stage 4: <30%), age group, gender (male vs. female), BMI class (I: <18.5 kg/m^2^, II: 18.5–24.99 kg/m^2^, III: 25–29.99 kg/m^2^, IV: 30–34.99 kg/m^2^, V: >35 kg/m^2^, smoking status (Group I: Active smoker vs. Group II: Former smoker/never smoker), number of COPD exacerbation one year prior to baseline (0 vs. 1 vs. >2) and comorbidities using Charlson Comorbidity Index [19]. Smoking status group I includes active smokers and former smokers with smoking cessation of less than 6 months prior to registration in DrCOPD. Smoking status group II includes never smokers and former smokers with smoking cessation of more than 6 months prior to registration in DrCOPD. Survival analysis for the propensity-matched population was conducted with unadjusted Cox proportional hazard models. A confidence interval of 95% (*p* < 0.05) was considered statistically significant.

For sensitivity analyses, we conducted adjusted Cox proportional hazard model in the unmatched population at 180-day follow-up and 365-day follow-up and in the matched population with follow-up period of 365 days. The Cox proportional hazard model on unmatched population was adjusted for the following confounding variables: GOLD stage 1–4, age group, gender (male vs. female), BMI class (Class I–V), smoking status (Group I: Active smoker vs. Group II: Former smoker/never smoker), number of COPD exacerbation one year prior to baseline (0 vs. 1 vs. >2) and Charlson Comorbidity Index.

A subgroup analysis was performed to determine the association between MTX doses and risk of AECOPD. Patients were grouped into three groups: (1) No MTX use, (2) Low dose MTX and (3) Medium/high dose MTX. Low dose group was defined as one MTX prescription within two years prior to study inclusion. Medium/high dose was defined as >1 MTX prescriptions within two years prior to study entry. One prescription is equivalent to 100 pills at 2.5 mg each; a total of 250 mg MTX.

Finally, control of Cox proportional hazard model was performed. The following criteria were reviewed: linearity of continuous variables, lack of drug interaction (OCS) and assumption of hazard proportionality (PHA) throughout study period.

Statistical analysis was performed using the SAS statistical software version 9.4 (SAS Institute, Cary, NC, USA).

## 3. Results

### 3.1. Descriptive Analyses

**Figure 1:**
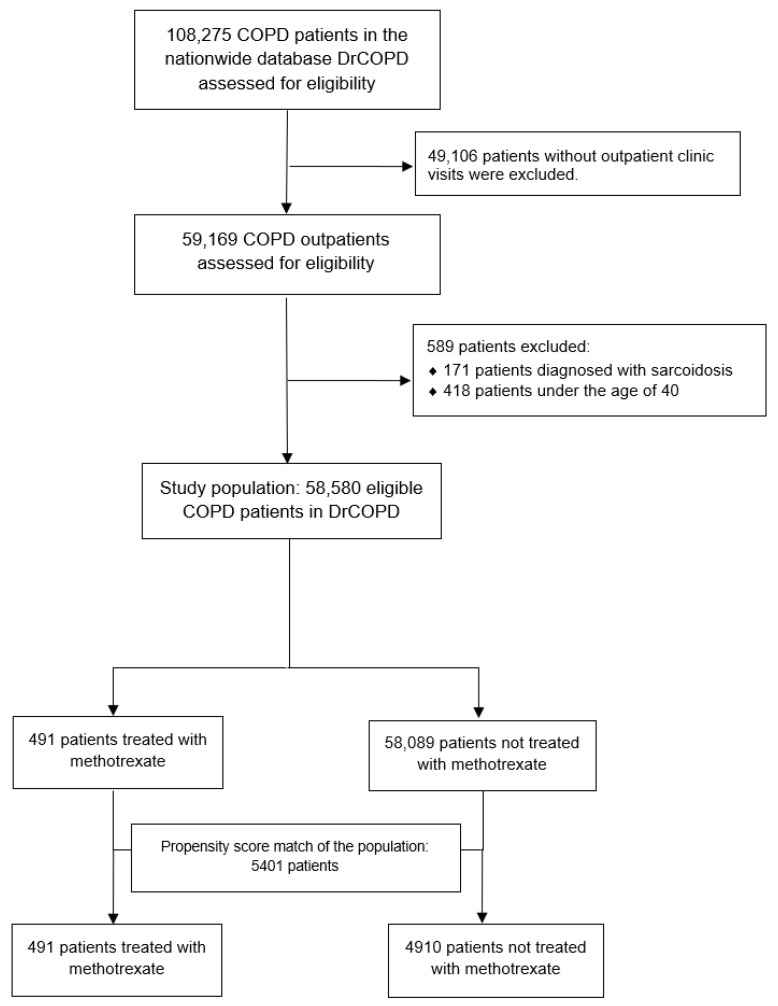
Study flowchart. 58,580 eligible patients registered with COPD in DrCOPD from 1 January 2010 to 31 December 2017 were included in our study. Abbreviations: COPD, Chronic Obstructive Pulmonary Disease; DrCOPD, The Danish Registry of Chronic Obstructive Pulmonary Disease.

The demographic and clinical characteristics for the study population is presented in Table 1. Of the 108,275 patients with COPD in the DrCOPD, 58,580 patients met all study inclusion criteria (Figure 1).

A total of 491 (0.84%) patients were identified as MTX users. Our study population consisted of 30,606 females and 27,974 males. The median age was 70 years (63–78). The number of patients who had hospitalization-requiring COPD exacerbation in the year leading up to study entry was 17,457. All prescriptions for LAMA, LABA and ICS (monotherapy and combination therapy) as well as accumulated doses of OCS dispensed 12 months prior to the date of study entry were identified. The matched population and the unmatched population were similar in baseline characteristics in terms of age group, BMI class, number of AECOPD 12 months prior to study inclusion, ICS use, LAMA and LABA use. There were slight differences in GOLD class, smoking status, OCS use and comorbidity index (Table 1).

### 3.2. Statistical Analysis

#### 3.2.1. Main Outcome Analysis

**Figure 2:**
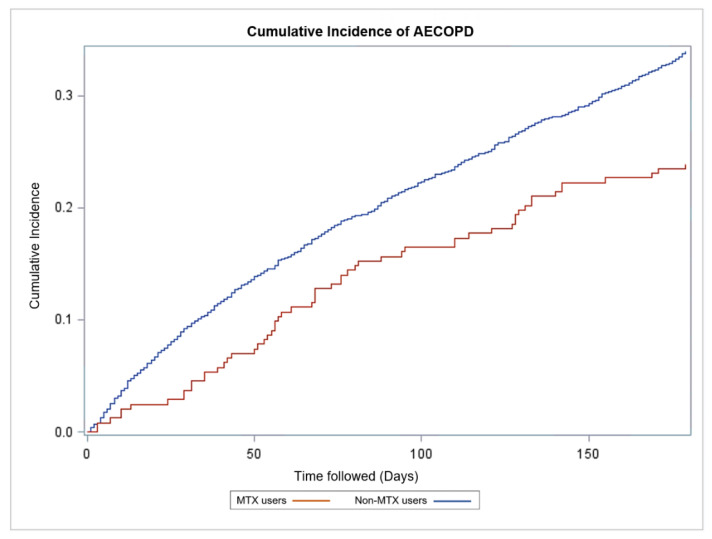
Cumulative incidence of AECOPD in MTX users and non-MTX users in the matched population at 180-day follow-up. MTX was associated with a markedly lower risk of hospitalization-requiring COPD exacerbation within the first half year after study entry (HR 0.66, CI 0.66–0.66, *p* < 0.001). Demographic and clinical characteristics of our study population. Abbreviations: COPD, Chronic Obstructive Pulmonary Disease; AECOPD, Acute hospitalization-requiring exacerbation of COPD; MTX, Methotrexate; HR, Hazard ratio; CI, Confidence interval.

The propensity scores matched population consisted of 5401 patients with COPD. There were 719 AECOPD events in the non-MTX group and 58 AECOPD events in the MTX group in the 180-day study period. The risk of AECOPD after 180 days of follow-up was lower for the MTX group compared to the non-MTX group (Hazard ratio (HR) 0.66, Confidence Interval (CI) 0.66–0.66, *p* < 0.001). Log-rank test showed a statistically significant difference in the cumulative incidence curves of the two study groups (*p* = 0.003) (Figure 2).

#### 3.2.2. Sensitivity Analysis

**Figure 3:**
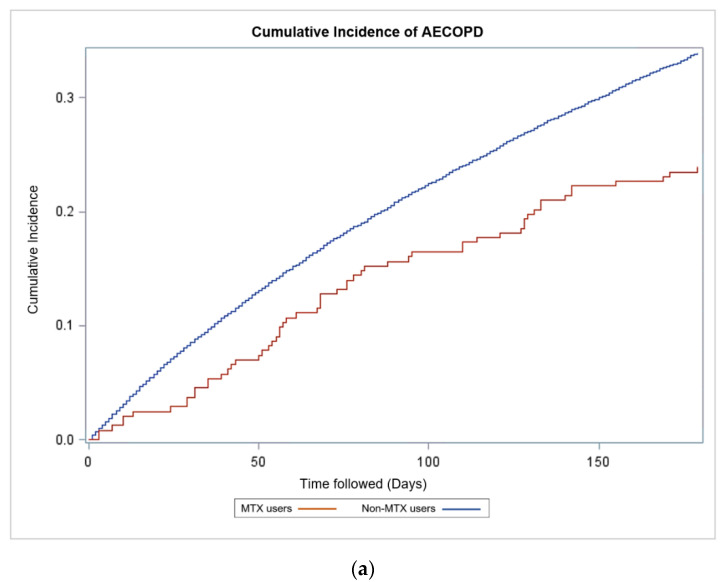

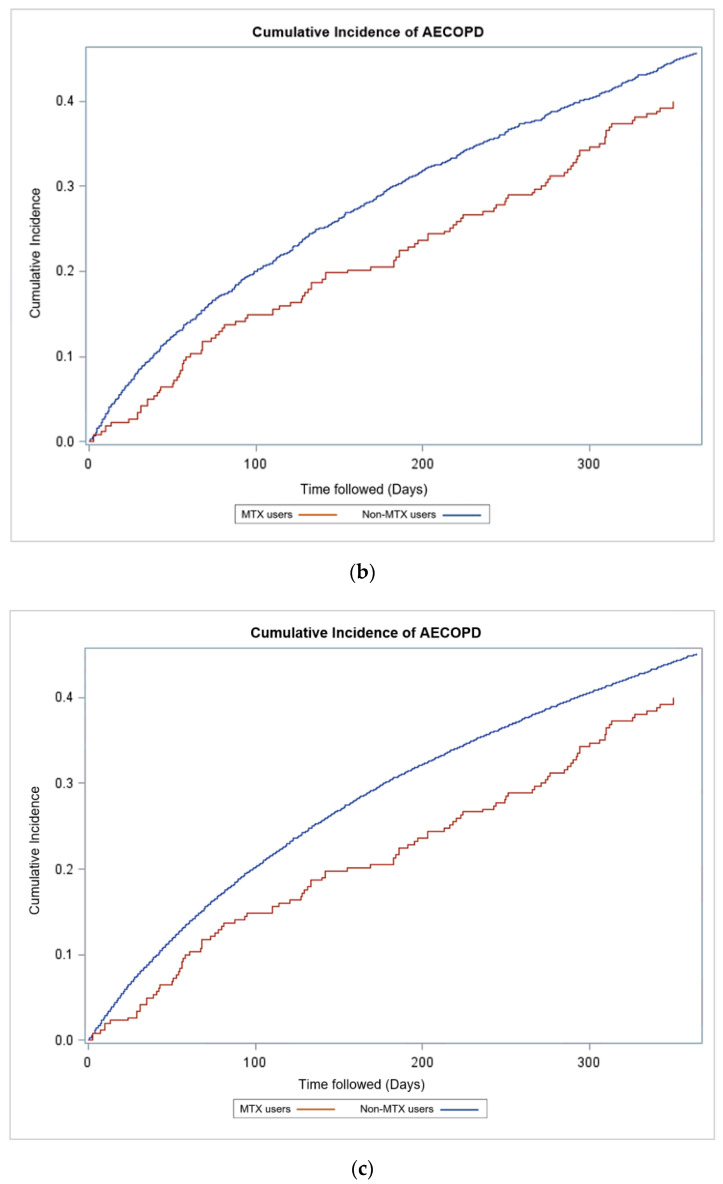
Sensitivity Analyses—Cumulative Incidence of AECOPD. (**a**) Cumulative incidence of AECOPD in MTX users and non-MTX users in the unmatched population at 180-day follow-up. MTX was associated with lower risk of hospitalization-requiring COPD exacerbation (HR 0.76, CI 0.59–0.99, *p* = 0.04); (**b**) Cumulative incidence of AECOPD in MTX users and non-MTX users in the matched population at 365-day follow-up. MTX was associated with lower risk of hospitalization-requiring COPD exacerbation (HR 0.81, CI 0.81–0.82, *p* < 0.001); (**c**) Cumulative incidence of AECOPD for MTX users and non-MTX users in the unmatched population at 365-day follow-up. MTX was associated with lower risk of hospitalization-requiring COPD exacerbation (HR 0.92, CI 0.76–1.11, *p* = 0.37). COPD, Chronic Obstructive Pulmonary Disease; AECOPD, Acute hospitalization-requiring exacerbation of COPD; MTX, Methotrexate; HR, Hazard ratio; CI, Confidence interval.

An adjusted Cox proportional hazard model was developed on the unmatched population at 180-day follow-up. There were 8577 AECOPD events in the non-MTX group and 58 AECOPD events in the MTX group. MTX treatment showed a reduction in risk of AECOPD (HR 0.76, CI 0.59–0.99, *p* = 0.04) (Figure 3a, Table 2). Similar result was observed in the analysis of matched population at 365-day follow-up 1057 AECOPD events in the non-MTX group and 105 AECOPD events in the MTX group. (HR 0.81, CI 0.81–0.82, *p* < 0.001) (Figure 3b, Table 2). In the unmatched population at 365-day follow-up, Cox proportional hazard model showed no significant effect on risk of AECOPD in MTX group compared to the non-MTX group (HR 0.92, CI 0.76–1.11, *p* = 0.37). Cumulative incidence curves for this analysis are illustrated in Figure 3c. As seen here, a greater cumulative incidence was observed in the non-MTX group.

Log-rank test showed statistically significant difference in the cumulative incidence curves for the two study groups in both the matched analysis at 365-day follow-up and unmatched analysis at 180-day follow-up (*p* = 0.045 and *p* = 0.002, respectively).

#### 3.2.3. Subgroup Analysis

There were 472 patients in the low dose MTX group and 19 patients in the medium/high dose MTX group. Subgroup analysis on risk of AECOPD in no MTX use group, low dose MTX group and medium/high dose MTX group in the propensity-matched population at 180-day follow-up showed that both the low dose MTX group and the medium/high dose MTX group were associated with lower risk of AECOPD compared to the no MTX use group. (Low dose: HR 0.61, CI 0.46–0.80, *p* = 0.0005; Medium/High dose: HR 0.54, CI 0.13–2.2, *p* = 0.40; No MTX use—HR reference).

## 4. Discussion

We found that the use of MTX for COPD outpatients was associated with a substantially lower risk of severe AECOPD or death within half a year, both in the main propensity-matched analysis and in an adjusted Cox regression including the whole population. Our sensitivity analyses at one year showed similar results, yet with a lesser pronounced signal. Our subgroup analysis also showed lower risk of AECOPD in both the low dose MTX and medium/high dose MTX with a more pronounced risk reduction in the medium/high dose MTX group. This could indicate an association between MTX doses and risk of AECOPD despite very few patients in the medium/high dose MTX group. Lower risk of AECOPD in COPD patients treated with MTX seems biologically plausible since AECOPD is often caused by an infection and MTX, with its ability to inhibit cell synthesis and hereby the proliferation of immune cells during an infection, is as an anti-inflammatory agent. This anti-inflammatory property could potentially have a prophylactic effect on COPD patients and reduce the risk of lung infection that may lead to AECOPD.

To our knowledge this is the first systematic analysis of MTX and the risk of severe COPD exacerbation. Surprisingly, close to no evidence exists on this area, although it is generally recognized that COPD exacerbation is an inflammatory condition and likewise that MTX is an anti-inflammatory drug, which can be corticosteroid-sparing for other inflammatory conditions. In asthma, a few small trials have explored the use of MTX, however, only as a mean to reduce the use of systemic corticosteroids [21,22], and no solid data exist on clinical outcomes for asthma patients using MTX in comparison to others not using this drug. In this meta-analysis (Davies et al.), a small reduction in systemic corticosteroids was found, however, at a cost of side effects, and the authors did not recommend using this treatment, except in selected cases.

This corticosteroid-sparing effect of MTX was further demonstrated in the treatment of sarcoidosis [14], dermatomyositis [23], thyroid eye disease [24], scleritis [25,26] and neurocysticercosis [27]. In randomized clinical trials (RCT), similar results were demonstrated in the treatment of juvenile dermatomyositis [28].

When administered correctly, MTX is well-tolerated in most autoimmune and chronic inflammatory disorders, but like other drugs, the use of MTX comes with potential adverse effects. The adverse effect of main concern in regarding to lung patients is direct damage to lung tissue and increased risk of opportunistic infections [12,29]. Despite the possibility of pulmonary-related adverse effects, studies on MTX treatment have shown minimal risk of pulmonary toxicity and such risk should be weighed against the serious adverse effects of corticosteroids [30].

### Strengths and Limitations

This study is to our knowledge the first study conducted to explore the impact of MTX on COPD patients. Strengths include the large study size of severely ill COPD-patients relatively well-characterized through the national COPD registry. Further, the outcomes explored are followed to almost full completeness from our national registries. This detailed knowledge on the status of several key predictors of prognosis in these patients minimizes the risk of bias by allowing us to control for several important factors. In addition, all patients in the cohort had a respiratory medicine specialist confirmed COPD diagnosis and this was validated at least annually, likewise by a respiratory medicine specialist, thus reducing the risk of misclassification bias.

Even though our study had some strengths, it also carried some limitations. First, the retrospective nature of data collection limited the ability infer causation. However, we did protocolize and web-publish the study design and plan prior to analysis. Second, although we did find MTX use in 491 patients, this could have been insufficient to assure enough power for subgroup analysis. Third, MTX users may, although they had more rheumatic comorbidity (indication), have been “healthy participants” selected to be able to tolerate MTX. Patients were propensity-score matched at a 1:10 ratio which increases precision, but at a cost of less accuracy in matches resulting in a comparison of outcome in groups of patients with varying risk levels [31].

Fourth, we adjusted our statistical analyses for known confounders, but residual confounding such as genetics and medical incompliance could not be accounted for. The Danish National Health Service Prescription Database provides information on drug dispensation, but whether the dispensed drugs are taken by the patients involves uncertainties and creates additional potential source of error alongside other unknown confounders. Fifth, our registries did not contain information on the weekly doses of MTX, the length of treatment, and cause of use for MTX making it not possible to fully assess whether the posology of the drug is associated with a protective effect on AECOPD and which minimum treatment duration is required for an effect to be observed.

MTX was strongly associated with lower risk of severe hospitalization-requiring AECOPD, and our results seemed consistent in the adjusted models, the unadjusted propensity-matched cohort and subgroup analyses. Likewise, the anti-inflammatory properties of MTX makes the clinical impact biologically plausible. Randomized trials should be performed to examine whether our findings are causative.

## Figures and Tables

**Table 1:** The demographic and clinical characteristics of our study population.

N (Number of Participants)	Entire COPD Cohort (*n* = 58,580)			Propensity-Matched Cohort (*n* = 5401)		
	Non MTX Group	MTX Group	*p*	Non MTX Group	MTX Group	*p*
Characteristics	*n* = 58,089	*n* = 491		*n* = 4910	*n* = 491	
Age, median (IQR)	70 (63–78)	69 (63–76)	<0.0001	70 (63–78)	69 (63–75)	<0.0001
Male (%)	27,787 (47.8)	187 (38.1)	<0.0001	2277 (46.4)	187 (38.1)	0.0004
FEV_1_(%) median (IQR)	49 (37–61)	51 (42–64)	<0.0001	49 (37–61)	52 (42–66)	<0.0001
GOLD stage 4: <30, *n* (%)	7615 (13.1)	34 (6.92)	<0.0001	637 (13.0)	34 (6.92)	<0.0001
GOLD stage 3: 30–49.99, *n* (%)	24,924 (42.9)	192 (39.1)		2137 (43.5)	192 (39.1)	
GOLD stage 2: 50–79.99, *n* (%)	22,009 (37.9)	219 (44.6)		1832 (37.3)	219 (44.6)	
GOLD stage 1: ≥80, *n* (%)	3541 (6.10)	46 (9.37)		304 (6.19)	46 (9.37)	
BMI (kg/m^2^)						
BMI; Median (IQR)	25 (22–28)	25 (22–29)	<0.0001	25 (22–28)	25 (22–29)	<0.0001
I: 10.0–18.4	5061 (8.71)	32 (6.52)	0.04	466 (9.49)	32 (6.52)	0.02
II: 18.5–24.9	20,363 (35.1)	154 (31.4)		1726 (35.2)	154 (31.4)	
III: 25.0–29.9	21,042 (36.2)	184 (37.5)		1740 (35.4)	184 (37.5)	
IV: 30.0–34.9	7595 (13.1)	80 (16.3)		632 (12.9)	80 (16.3)	
V: ≥35	4028 (6.93)	41 (8.35)		346 (7.05)	41 (8.35)	
Smoking Status						
Active smoker (%)	19,294 (33.2)	132 (26.9)	0.003	1637 (33.3)	132 (26.9)	0.004
Former smoker/never-smoker (%)	38,795 (66.8)	359 (73.1)		3273 (66.7)	359 (73.1)	
AECOPD 12 months prior to study entry						
0 AECOPD (%)	40,778 (70.2)	345 (70.3)	0.83	3485 (71.0)	345 (70.3)	0.61
1 AECOPD (%)	8051 (13.9)	69 (14.1)		652 (13.3)	69 (14.1)	
≥2 AECOPD (%)	9260 (15.8)	77 (15.7)		773 (15.7)	77 (15.7)	
Use of ICS 12 months prior to study entry, *n* (%)	48,299 (83.2)	406 (82.7)	0.79	4083 (83.2)	406 (82.7)	0.79
Use of LAMA 12 months prior to study entry, *n* (%)	50,171 (86.4)	409 (83.3)	0.049	4251 (86.6)	409 (83.3)	0.044
Use of LABA 12 months prior to study entry, *n* (%)	51,823 (89.2)	429 (87.4)	0.043	4398 (89.6)	429 (87.4)	0.039
Use of OCS 12 months prior to study entry, *n* (%) ^a^						
No OCS use	34,314 (60.2)	213 (43.4)	<0.0001	2979 (60.7)	213 (43.4)	<0.0001
Low OCS use	10,927 (19.2)	85 (17.3)		929 (18.9)	85 (17.3)	
Medium/High OCS use	11,722 (20.6)	193 (39.3)		1002 (20.4)	193 (39.3)	
Accumulated OCS dose in mg 12 months prior to study entry, median (IQR)	1000 (250–2750)	1500 (750–2500)	<0.0001	875 (250–2500)	1500 (750–2500)	<0.0001
Use of MTX within 24 months prior to study entry, *n* (%) ^b^						
No MTX use	58,089	0	<0.0001	4910	0	<0.0001
Low MTX use	0	472 (96.1)		0	472 (96.1)	
Medium/High MTX use	0	19 (3.9)		0	19 (3.9)	
Astma diagnosis	8156 (14.0)	76 (15.2)	0.36	710 (14.5)	76 (15.2)	0.54
Charlson comorbidity index score ^c^, *n* (%)						
0	21,725 (37.4)	156 (31.8)	0.04	1877 (38.2)	156 (31.8)	0.04
1	12,585 (21.7)	112 (22.8)		1083 (22.1)	112 (22.8)	
≥2	23,779 (40.9)	223 (45.4)		1950 (39.7)	223 (45.4)	

Abbreviations: COPD, chronic obstructive pulmonary disease; IQR, interquartile range; FEV_1_, forced expiratory volume in 1 s; GOLD, Global Initiative for Obstructive Lung Disease; BMI, body mass index; AECOPD, acute hospitalization-requiring COPD exacerbation; ICS, inhaled corticosteroids; LAMA, long-acting muscarinic antagonist; OCS, Oral corticosteroids. ^a^ Low, Medium and High are defined as accumulated OCS doses 365 days before study entry converted to mean daily doses in milligrams: Low: 0.01–1.99 mg mean daily dose; Medium/High: ≥2 mg mean daily dose. ^b^ Low dose group is defined as one MTX prescription within two years prior to study inclusion. Medium/high dose is defined as >1 MTX prescriptions within 2 years prior to study entry. One prescription is equivalent to 100 pills at 2.5 mg each; a total of 250 mg MTX. ^c^ Based on the following comorbidities: heart failure, ischemic heart disease, diabetes, peptic ulcer, dementia, solid metastatic tumor, hemiplegia or paraplegia, renal failure, peripheral vascular disease, cerebrovascular dis-ease, rheumatic disease, any malignancy except malignant neoplasm of skin, mild liver disease and moderate to serious liver disease. Chronic pulmonary diseases were not included since all patients have COPD. AIDS/HIV infection was also not included, as it is not considered to decrease life expectancy if treated nowadays [20].

**Table 2:** Risk of AECOPD for the propensity-matched population and the unmatched population.

	AECOPD Event		Risk of AECOPD		
	MTX Group	Non-MTX Group	HR	(95% CI)	*p*-Value
Main Analysis					
Propensity-matched population, 180 days	58	719	0.66	0.66–0.66	<0.001
Sensitivity Analysis					
Unmatched population, 180 days	58	8577	0.76	0.59–0.99	0.041
Propensity-matched population, 365 days	105	1057	0.81	0.81–0.82	<0.001
Unmatched population, 365 days	105	12,399	0.92	0.76–1.11	0.37

Abbreviations: COPD, chronic obstructive pulmonary disease; AECOPD, Acute hospitalization-requiring exacerbation of COPD; MTX, Methotrexate; HR, Hazard ratio; CI, Confidence interval.

## Data Availability

We believe that knowledge sharing increases the quantity and quality of scientific results. Sharing of relevant data will be discussed within the study group upon reasonable request.

## Supplementary Materials

The following are available online at https://www.mdpi.com/article/10.3390/biomedicines9060604/s1, Table S1: Medications from The Danish National Health Service Prescription Database identified using ATC codes, Table S2: Diagnoses identified using ICD-10 codes from The Danish National Patient Register.
