# Supplementary material for: Risk of Chronic Obstructive Pulmonary Disease Exacerbation in Patients Who Use Methotrexate—A Nationwide Study of 58,580 Outpatients
Source: Biomedicines. 2021 May 26;9(6):604. doi: 10.3390/biomedicines9060604 (PMC8229017; doi:10.3390/biomedicines9060604)
Supplement: Supplementary file 1 [file biomedicines-09-00604-s001.zip › biomedicines-1191950-supplementary.pdf]

Supplementary Materials

Table S1. Medications from The Danish National Health Service Prescription Database identified using ATC codes.

Medication	ATC-Code
MTX	L01BA01, L04AX03
ICS	R03BA01, R03BA02, R03BA03, R03BA05, R03BA07, R03BA08
ICS/LABA combination therapy	R03AK06, R03AK07, R03AK08, R03AK10, R03AK11, R03AK14
ICS/LAMA/LABA combination therapy	R03AL08, R03AL09, R03AL12
LAMA	R03BB01, R03BB02, R03BB04, R03BB05, R03BB06, R03BB07
LAMA/LABA combination therapy	R03AL05, R03AL07, R03AL04, R03AL06, R03AL03
LABA	R03AC12, R03AC13, R03AC18, R03AC19
OCS	H02AB06

Abbreviations: MTX, Methotrexate; ICS, Inhaled Corticosteroids; LABA, long-acting beta-agonist; LAMA, long-acting muscarinic antagonist; OCS, Oral Corticosteroids

Table S2: Diagnoses identified using ICD-10 codes from The Danish National Patient Register.

**Diagnosis**	**ICD-10 Code**
Sarcoidosis	D86
Heart failure	I50
Ischemic heart disease	I21–I25
Diabetes	E10–E14
Peptic ulcer	K25–K28
Dementia	F00 – F03
Solid, metastatic tumour	C77–C80
Hemiplegia or paraplegia	G80–G83
Renal failure	I120, I131, I132, E102, E112, E122, E132, E142, N02-N08, N11, N14, N150, N158 – N165, N168, N169, N18, N19, N26, Z992
Peripheral vascular disease	I70 – I74, I77, I79
Cerebrovascular disease	I60 – I64, I67, G45, G46
Rheumatic disease	M05, M06, M32–M36
Malignant tumours	C00–C26, C30–C34, C37–C41, C45–C58, C60–C76, C81–C85, C90–C97, C43, C88
Mild liver disease	B18, K73, K74, K717, K768, K769, K760, K709, K700–K703, K713–K715, K762–K764
Moderate to severe liver disease	I850, I859, I864, I982, K704, K711, K721, K729, K765, K766, K767
AECOPD	J12–J18, J20–J22, J40–J44, J96

Abbreviations: AECOPD, acute hospitalization-requiring exacerbation of COPD
